# Editorial: The role of high-order chromatin organization in gene regulation

**DOI:** 10.3389/fgene.2022.1045787

**Published:** 2022-10-03

**Authors:** Veniamin Fishman, Alexey V Pindyurin

**Affiliations:** ^1^ Institute of Cytology and Genetics SB RAS, Novosibirsk, Russia; ^2^ Novosibirsk State University, Novosibirsk, Russia; ^3^ Institute of Molecular and Cellular Biology SB RAS, Novosibirsk, Russia

**Keywords:** Hi-C, chromatin architecture, genomics, nuclei, transcription regulation, 3C

It is now accepted that high-order chromatin packaging is non-random and essential for genome functioning. However, details of mechanisms underlying chromatin folding and its role in epigenetic regulation remain to be discovered. One of the most actively studied problems in this field is understanding the molecular basis of correlations between gene expression and chromatin architecture. Although causal relationships between these processes are still not well established, information about genome folding can already be employed for functional interpretation of genomic variants. For example, spatial proximity between gene promoters and regulatory elements can be instructive for linking GWAS variants with their putative target genes. An article by Thiecke et al. published in this Research Topic describes how information about genome folding was employed for prioritization of candidate genes underpinning COVID-19 host genetic traits. Similarly, chromatin architecture can help to understand cancer genomics. Studies by Adeel et al. and Baur et al. groups show how to infer connections between genome architecture and disease susceptibility by profiling chromatin contacts in multiple cancer samples. This approach allows authors to identify chromatin alterations specific for cervical and breast cancer development.

Several papers published in this Research Topic allow more comprehensive review of known mechanisms and new hypothesis explaining connections between changes of chromatin architecture. The paper by Boltsis et al. published in this article Research Topic, describes general principles connecting genome architecture with development and disease. Another review by Daghsni and Aldiri discusses this problem in the context of mammalian retina development, and reviews Tchurikov and Kravatsky and Malashicheva and Perepelina highlights the important role of ribosomal genes clusters and lamin proteins in global epigenetic regulation. Finally, a review by Kumar et al. extends the subject to the area of plant genomics.

Articles mentioned above show the importance of studying chromatin architecture in cancer and other human tissues. However, in many experimental systems, application of conventional chromatin profiling techniques is challenging due to a limited amount and/or peculiar properties of the available biological material. Several articles in this Research Topic describe new methodologies aiming to overcome this limitation. Research by Animesh et al. shows how to apply Hi-C method to nasopharyngeal cancer needle biopsy patient samples. In the article by Bylino et al. perform critical analysis of the chromosome conformation capture procedure to provide a useful guide to the 3C procedure. Another research from the same group Bylino et al. describes application of this updated 3C method to study the developmental genes in *Drosophila* larvae. Finally, Aljogol et al. compare computational methods of capture Hi-C-data analysis and highlight their advantages and disadvantages.

Whereas some of the aforementioned methods aim to modify the original Hi-C protocol to make it suitable for low-input samples, a very important direction of research is single-cell analysis of genome folding. Complementary to these new molecular methods, recently developed high-resolution microscopy approaches extend our understanding of genome architecture in individual cells. Cardozo Gizzi discusses how new molecular technologies developed in the rapidly evolving single-cell genomics field change our view of chromatin architecture. And two articles Maslova and Krasikova and Zakirov et al., discuss how the structures revealed by molecular and modern microscopy analysis correspond to each other.

Overall, this article Research Topic shows how using new computational and molecular tools can extend our understanding of mechanisms underlying chromatin folding and transcription dynamics, and how this knowledge can be used in solving problems in fundamental biology, agriculture, and molecular medicine.

